# Variation in the Distribution of Putative Virulence and Colonization Factors in Shiga Toxin-Producing *Escherichia coli* Isolated from Different Categories of Cattle

**DOI:** 10.3389/fcimb.2017.00147

**Published:** 2017-04-28

**Authors:** María E. Cáceres, Analía I. Etcheverría, Daniel Fernández, Edgardo M. Rodríguez, Nora L. Padola

**Affiliations:** ^1^Laboratorio de Inmunoquímica y Biotecnología, Departamento de Sanidad Animal y Medicina Preventiva, Centro de Investigación Veterinaria Tandil, CONICET, CICPBATandil, Argentina; ^2^Laboratorio de Inmunoquímica y Biotecnología, Departamento de Sanidad Animal y Medicina Preventiva, Facultad de Ciencias VeterinariasTandil, Argentina; ^3^Área de Bioestadística, Departamento Sanidad Animal y Medicina Preventiva, Centro de Investigación Veterinaria, Facultad de Ciencias VeterinariasTandil, Argentina

**Keywords:** shiga toxin-producing *Escherichia coli*, category of cattle, plasmid genes, adhesins, production systems

## Abstract

Shiga toxin-producing *Escherichia coli* (STEC) are pathogens of significant public health concern. Several studies have confirmed that cattle are the main reservoir of STEC in Argentina and other countries. Although Shiga toxins represent the primary virulence factors of STEC, the adherence and colonization of the gut are also important in the pathogenesis of the bacteria. The aim of this study was to analyze and to compare the presence of putative virulence factors codified in plasmid -*katP, espP, subA, stcE*- and adhesins involved in colonization of cattle -*efa1, iha*- in 255 native STEC strains isolated from different categories of cattle from different production systems. The most prevalent gene in all strains was *espP*, and the less prevalent was *stcE*. *katP* was highly detected in strains isolated from young and rearing calves (33.3%), while *subA* was predominant in those isolated from adults (71.21%). Strains from young calves showed the highest percentage of *efa1* (72.46%), while *iha* showed a high distribution in strains from rearing calves and adults (87.04 and 98.48% respectively). It was observed that *espP* and *iha* were widely distributed throughout all strains, whereas *katP, stcE*, and *efa1* were more associated with the presence of *eae* and *subA* with the *eae*-negative strains. A great proportion of *eae*-negative strains were isolated from adults -dairy and grazing farms- and from rearing calves -dairy and feedlot-, while mostly of the *eae*-positive strains were isolated from dairy young calves. Data exposed indicate a correlation between the category of the animal and the production systems with the presence or absence of several genes implicated in adherence and virulence of STEC.

## Introduction

Shiga toxin-producing *Escherichia coli* (STEC) are endemic pathogens in Argentina with a high impact on the health system. STEC has been associated with outbreaks and sporadic cases of human disease, causing diarrhea, hemorrhagic colitis (HC) and hemolytic uremic syndrome (HUS) (Paton and Paton, 1998).

The primary virulence factors of STEC are Shiga toxins (*stx*), however the adherence and colonization of gut are also important and constitute the first step in the pathogenesis of the bacteria (Szalo et al., 2002). Another typical virulence factor is Intimin, an outer membrane adherence protein encoded by *eae*, located in the locus of enterocyte effacement (LEE), which is responsible for the histopathological lesion called “attaching and effacing” (A/E) (Nataro and Kaper, 1998). The LEE-pathogenicity island is present in a set of STEC serotypes considered to be highly virulent, however, LEE not appear to be essential for pathogenesis, since a large number of LEE-negative strains has been associated with sporadic outbreaks of HC and HUS (Cergole-Novella et al., 2007; Cundon et al., 2015). Other colonization factors beyond Intimin have been described: An adherence-conferring protein similar to the Vibrio cholerae IrgA (*iha*) that confer the capacity to adhere to epithelial cells in a diffuse pattern (Tarr et al., 2000) and an enterohemorrhagic *E. coli* factor of adherence (*efa1*) implicated in the intestinal colonization of calves (Nicholls et al., 2000; Stevens et al., 2002).

There are additional putative virulence factors encoded by plasmids that contribute to the survival and citotoxicity of STEC, such as an hemolysin (*ehxA*) encoded by the plasmid pO157 with cytotoxic activity in human and bovine cell lines (Schmidt et al., 1995), a katalase-peroxidase (*katP*) which is part of bacterial defense mechanisms against oxidative stress, an extracellular serine protease (*espP*) that is able to cleave pepsin and coagulation factor V, suggesting that it might be an accessory virulence factor exacerbating HC (Caprioli et al., 2005; Brockmeyer et al., 2007), and a zink metalloprotease (*stcE*), which modify the protective layer of the host intestine to assist the pedestal-actine formation encoded by LEE contributing to intimate adherence to cells (Grys et al., 2005). The presence of other toxins in addition to Stx has been reported, such as the subtilase cytotoxin (*subAB*) encoded by the plasmid pO113 particulary carried by *eae*-negative STEC strains. This toxin is lethal for mice, inhibit protein synthesis, have pro-inflammatory properties, and may play a significant role in pathogenesis of STEC disease (Paton and Paton, 2010).

Several studies have confirmed that cattle are the main reservoir of STEC O157 and non-O157 serotypes in Argentina and other countries, with variable prevalence ranged from 22 to 62.7% in different categories of cattle (Sanz et al., 1998; Blanco et al., 2004; Padola et al., 2004). In young cattle, a prevalence of STEC was showed in 25, 43, and 58% in newborn calves, milk-fed, and growing dairy calves, respectively. The presence of STEC in newborn calves <24 h old suggested that they are exposed to this bacterium quickly after birth, which migth play an important role in vertical STEC transmission (Fernández et al., 2012). Also, a high prevalence of STEC in dairy cows, preslaughter and healthy grazing cattle -37.5, 44, and 22%, respectively- has been detected (Sanz et al., 1998; Fernández et al., 2009). Recent studies confirm the age of cattle as an important factor that significantly influences the shed of STEC, more than other physiological factors, such as breed, sex or weight gain. Data revealed that calves were more likely to shed STEC during the first 6 months, peaked at 2 years of age and declines as the animal matured. It was found that diversity of gut microflora could be a reason for that variation (Mir et al., 2014, 2016).

Contact with feces of cattle, direct contact with the animals or their environment and consumption of contaminated beef, milk, dairy products, water, unpasteurized apple juices, and vegetables, are possible routes for STEC human exposure and disease (Cody et al., 1999; Olsen et al., 2002; Jure et al., 2010; Brusa et al., 2013). Because the most of the STEC human infections are caused by consumption of contaminated animal products, the knowledge of the virulence profiles and colonization factors of STEC among different animals categories could provides information to evaluate some strategies to control cattle shedding in order to decrease the incidence of disease in humans. Therefore, the aim of this study was to analyze and to compare the presence of putative virulence factors codified in megaplasmid and adhesins involved in colonization of cattle in a collection of STEC strains isolated from different cattle categories and different production systems of Argentina.

## Materials and methods

### Bacterial isolates

A total of 255 *ehxA*-positive STEC strains from the Laboratorio de Inmunoquímica y Biotecnología (UNCPBA, Tandil, Buenos Aires, Argentina) were analyzed. All STEC strains were previously isolated from fecal samples of cattle from different categories, named in this study as: young calves (0–2 mo), rearing calves (2–8 mo) and adults (>8 mo), which came from different regional production systems (dairy farm, feedlot and grazing). STEC strains were serotyped and characterized for detection of typical virulence factors -*stx1, stx2, eae, ehxA*- and *saa* (Sanz et al., 1998; Padola et al., 2004; Fernández et al., 2010, 2012).

### PCR

For the detection of megaplasmid genes -*katP, espP, subA, stcE*- a multiplex PCR was performed according to the methodology described by Bustamante et al. (2011). Two PCRs were performed for the detection of *efa1* and *iha* according to that described by Nicholls et al. (2000) and Szalo et al. (2002), with some modifications. Primers and size of PCR products are shown in Table 1. PCR products were visualized by agarose gel (2%) electrophoresis, with ethidium bromide staining.

**Table 1:** **Characteristics of the PCR primers used in this study**.

**Primer**	**Primer secuence (5′ → 3′)**	**Target gene**	**Size of PCR amplicon (pb)**	**References**
*katP Fw*	GCGCCAGTGGTGGTCAGCAA	*katP*	914	Bustamante et al., 2011
*katP Rv*	ATATCGGGCTGCCGGTCCCA			
*espP Fw*	GCTGGCAACCAGCAACAGCG	*espP*	774	Bustamante et al., 2011
*espP Rv*	CGGTAGCCCGCTTCTGCACC			
*subHCDF*	TATGGCTTCCCTCATTGCC	*subA*	556	Paton and Paton, 2005
*subSCDR*	TATAGCTGTTGCTTCTGACG			
*stcE Fw*	GGCTCCGGAGGTGGGGGAAT	*stcE*	399	Bustamante et al., 2011
*stcE Rv*	GAAGCCGGTGGAGGAACGGC			
*iha Fw*	CAAATGGCTCTCTTCCGTCAATGC	*iha*	925	Szalo et al., 2002
*iha Rv*	CAGGTCGGGGTTACCAAGT			
88T14	GAGACTGCCAGAGAAAG	*efa1*	479	Nicholls et al., 2000
88T9	GGTATTGTTGCATGTTCAG			

### Statistical analysis

In order to describe the distribution of plasmid genes and adhesins between different categories of cattle, data were analyzed using the Chi2 test, or Fisher's exact test if necessary and possible, using the PROC FREQ procedure of Statistical Analysis Systems, Version 9.3 (SAS Institute, Cary, NY).

## Results

### Distribution of plasmid and adhesins genes in STEC according the category of cattle

**Figure 1:**
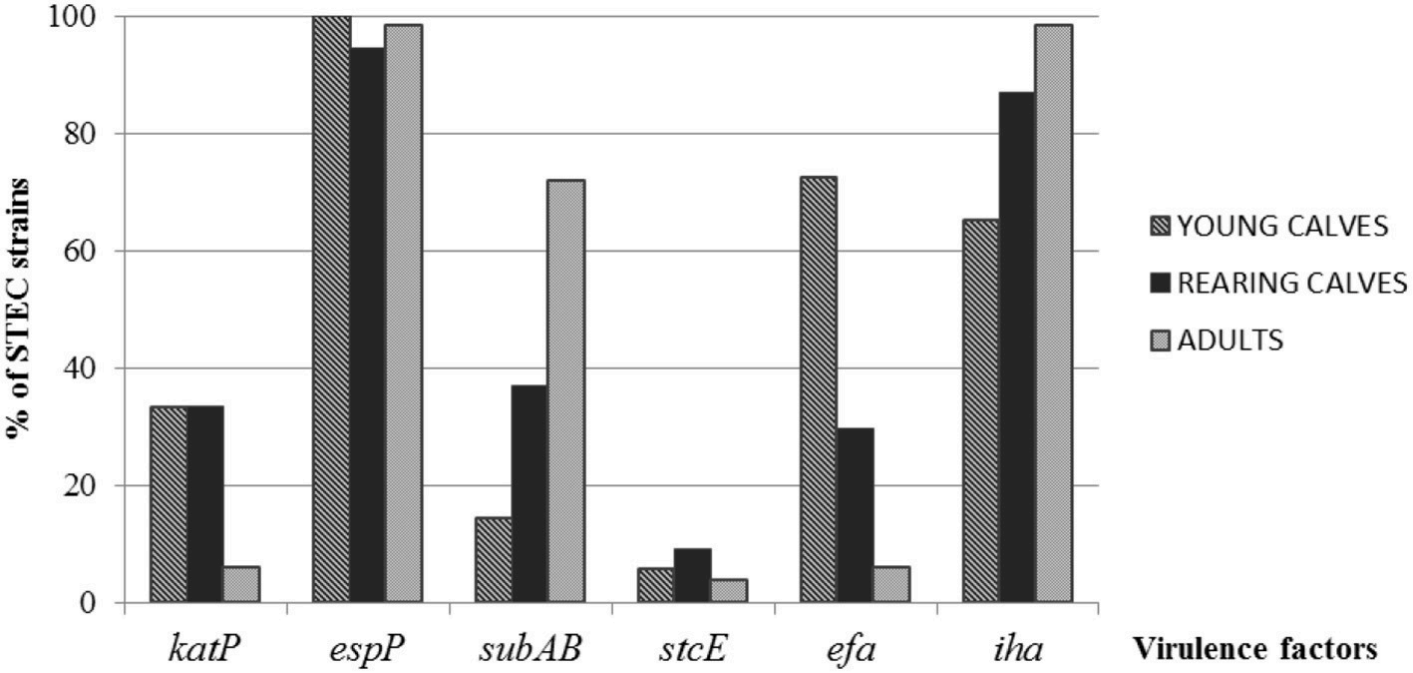
**Distribution of virulence factors in STEC strains among the different categories of cattle**.

The most prevalent gene detected among all strains regardless the category of cattle was *espP*, while *stcE* was the less prevalent (*p* = 0.0679 and *p* = 0.2670, respectively). For *katP*, the differences observed between categories, young calves, rearing calves and adults (33.3, 33.3, and 6.1%, respectively) were statistically significant (*p* < 0.0001). *SubA* was mostly found in strains isolated from adults (71.21%; *p* < 0.0001; Figure 1).

Significant differences in the distribution of *efa1* were observed, with a greater percentage in strains from young calves (72.46%) than rearing calves (29.63%) and adults (6.06%) (*p* < 0.0001). The ditribution of *iha* showed the highest proportion in strains isolated from adults (98.48%; *p* = 0.0126; Figure 1).

The most prevalent virulence profile in STEC strains isolated from young calves was *espP, efa1, iha* (21.73%), while *espP, subA, iha* was the most prevalent in rearing calves and adults (37 and 68.3% respectively) (Table 2).

**Table 2:** **Virulence profile carried by STEC clasified according to the category of cattle**.

**Category of cattle**	**Virulence profiles**	**No. (%) strains**
Young calves (*n* = 69)	*espP, efa1, iha*	15 (21.73)
	*katP, espP, efa1, iha*	10 (14.5)
	*espP, efa1*	10 (14.5)
	*katP, espP, efa1*	8 (11.6)
	*espP, subA, iha*	5 (7.2)
	*espP, iha*	4
	*espP, iha*	2
	*katP, espP, stcE, efa1, iha*	2
	*katP, espP, iha*	2
	*espP, subA, iha*	2
	*espP*	2
	*katP, espP, efa1*	1
	*espP, subA, efa1, iha*	1
	*espP, subA, efa1*	1
	*espP, subA*,	1
	*espP, stcE, efa1, iha*	1
	*espP, stcE*	1
	*espP, efa1*	1
Rearing calves (*n* = 54)	*espP, subA, iha*	20 (37)
	*katP, espP, efa1, iha*	6 (11)
	*katP, espP, iha*	5 (9.2)
	*espP*	5 (9.2)
	*espP, iha*	4 (7.4)
	*katP, espP, stcE, efa1, iha*	3
	*espP, efa1, iha*	2
	*katP, espP, stcE, iha*	2
	*efa1, iha*	2
	*katP, espP, stcE, efa1*	1
	*espP, iha*	1
	*katP, espP, efa1, iha*	1
	*espP, ehxA, efa1*	1
	*iha*	1
Adults (*n* = 132)	*espP, subA, iha*	91 (68.3)
	*espP, iha*	25 (19)
	*katP, espP, stcE, efa1, iha*	3 (2.2)
	*katP, espP, efa1, iha*	2
	*katP, espP, iha*	2
	*espP, subA, efa1, iha*	2
	*espP, subA*	2
	*iha*	2
	*espP, efa1, iha*	1
	*katP, espP, stcE, iha*	1
	*espP, stcE, iha*	1

### Comparison of genes distribution among strains according presence or ausence of *eae*

Most *eae*-positive strains were isolated from young calves from dairy farm and rearing calves from feedlot; while *eae*-negative serotypes were isolated mostly from dairy and grazing adults. *katP, efa1*, and *stcE* were predominant in *eae*-positive serotypes while *subA* and *iha* were prevalent in *eae*-negative serotypes (Table 3).

**Table 3:** **Virulence profiles in STEC strain according to presence or absence of *eae***.

***eae* + (*n* = 89)**			***eae* − (*n* = 166)**		
**Virulence profiles**	**No. of strains**	**Serotypes**	**Virulence profiles**	**No. of strains**	**Serotypes**
*espP, efa1, iha*	18	O5:NM, O8:H16, O8:H25, O20:HNT, O26:H11, O38:H39, O103:NM, O103:H2/H8/H18, O111:NM, O113:H2, O118:H16, O145:NM, O146:NM, O153:H25, O157:H7, O165:NM, O171:H25, O172:NM/H21, NT	*espP, subA, iha*	116	O2:H5, O8:H19/H20, O20:H19, O27:H21, O37:H10, O39:H49, O46:H11, O41:H38, O55:NM, O64:NM, O74:H28, O79:NM, O88:H25, O91:H8/H21/H28, O103:H21, O105:H18, O113:H2/H21, O116:H21, O130:H11, O141:H7/H8, O153:H21/H25, O163:H19/H21, O174: H21, O178:H2/H7/H8/H19/H25/H28/H29, O179:NM, ONT:H7/H11/H46, NT, Autoaglutinante.
*katP, espP, efa1, iha*	18		*espP, iha*	31	
*espP, efa1*	10		*espP*	5	
*katP, espP, efa1*	9		*iha*	2	
*katP, espP, stcE, efa1, iha*	8		*espP, subA*	2	
*katP, espP, iha*	7		*espP, subA, efa1, iha*	2	
*espP, iha*	5		*katP, espP, iha*	2	
*espP*	2		*espP, efa1*	1	
*espP, subA, iha*	2		*espP, stcE, iha*	1	
*katP, espP, stcE, iha*	2		*katP, espP, stcE, iha*	1	
*efa1, iha*	2		*katP, espP, efa1*	1	
*espP, subA*	1		*katP, espP, efa1, iha*	1	
*espP, subA, efa1*	1				
*espP, subA, efa1, iha*	1				
*espP, stcE*	1				
*iha*	1				

## Discussion

Since that cattle has been recognized as the main reservoir of STEC, several strains from different sources and production systems have been isolated by our research group (Sanz et al., 1998; Parma et al., 2000; Padola et al., 2004; Fernández et al., 2009, 2010, 2012). The isolation and characterization of STEC from cattle is essential for the development of diagnostic and control tools to avoid the transmission of STEC to humans through the consumption of bovine derived contaminated food. In recent studies, the prevalence of STEC in beef cattle were investigated and a strong correlation between age and shedding was found (Mir et al., 2014). Calves have been identified as the major excretors of STEC decreasing with the growth of the animal. Similarly, the presence of *stx* has been correlated to age, specially *stx2*, being detected in young calves. Genotyping of additional factors in STEC isolated from animals with different ages is necessary for a more appropriate knowledge of their risk for human health (Mir et al., 2016).

In this study, the age as a factor of significant influence in the distribution of some STEC virulence and colonization factors was demonstrated. In agreement with Bustamante et al. (2011) we found a higher prevalence of *espP* among all STEC strains regardless the category of cattle and the production system, and *stcE* was the less prevalent. While *espP* was widely detected among *eae*-positives and *eae*-negatives serotypes, *stcE* was detected in a few *eae*-positive serotypes, such as O157:H7, O64:NM and NT. To our knowledge, this is the first report of the presence of *stcE* in O64:NM. A higher prevalence of *katP* in STEC isolated from young and rearing calves was found. All of the *katP*-positive strains also contained *espP*, and some of them harbored *stcE*.

Subtilase cytotoxin (SubAB) was firstly described in STEC O113:H21 responsible of an outbreak of HUS (Paton et al., 1999). Then, *subA* gene was indentified in various *eae*-negative STEC serotypes isolated from cattle, food and human patients (Cergole-Novella et al., 2007; Galli et al., 2010; Wu et al., 2010). In this study, *eae*-negative STEC serotypes O113:H21, O116:H21, O130:H11, O178:H19, and ONT:H7 carried *subA* and were isolated mostly from rearing calves and adults from dairy and grazing systems and, in agreement with Bustamante et al. (2011), none of *subA*-positive STEC harbored neither *stcE* nor *katP*.

Some putative adhesins have been described to inquire the mechanisms underlying the adherence of STEC strains to epithelial cells. In this study, wide differences between cattle categories were found regarding the adhesins investigated. In those STEC strains isolated from dairy young calves and feedlot rearing calves, high percentages of *efa1* were detected, declining with the increasing of age. In agreement with previous reports (Nicholls et al., 2000; Toma et al., 2004; Galli et al., 2010) the 89.5% of the *efa1*-positive strains were also *eae*-positive. Several authors have detected a high prevalence of *iha* in STEC strains isolated from animals, food, and humans, most of them *eae*-negative (Cergole-Novella et al., 2007; Galli et al., 2010). Our results show that *iha* was detected in all of STEC strains regarding the category of cattle, although it was more prevalent in those isolated from adults, and in *eae*-negatives STEC serotypes. However, Kobayashi et al. (2013) found high percentages of *iha* in *eae*-positives STEC strains, suggesting that this gene could have a wide distribution in STEC beyond the presence of *eae*.

Differences in genetic characteristics of STEC strains in reference to age may be explained by gut diversity microflora composition, low concentration of normal *Enterobacteriaceae* microflora that should influence STEC survival, adherence and colonization in the cattle intestine (Zhao et al., 2013). In adults animals, prevalence of STEC decrease while the commensal microflora increase its diversity, possibly influenced by diet of the animals which changes as the animal weans, grows and begins to incorpore pasture, or specific diet according the production system (Mir et al., 2016).

Data exposed indicate a correlation between the category of the animal and the production systems with the presence or absence of several genes implicated in adherence and virulence of STEC. Further studies are necessary to better understand the involvement of these genes in the dynamic of colonization and survival of this pathogen in cattle in order to establish control strategies to reduce the transmission of STEC from animals to humans.

## Author contributions

MC designed and performed the experiments, analyzed the data, wrote the paper. DF performed the experiments, revised the manuscript. ER analyzed statistically the data. AE and NP designed the experiments and revised critically the manuscript.

### Conflict of interest statement

The authors declare that the research was conducted in the absence of any commercial or financial relationships that could be construed as a potential conflict of interest.
